# Control of Diapause by Acidic pH and Ammonium Accumulation in the Hemolymph of Antarctic Copepods 

**DOI:** 10.1371/journal.pone.0077498

**Published:** 2013-10-15

**Authors:** Sabine Schründer, Sigrid B. Schnack-Schiel, Holger Auel, Franz Josef Sartoris

**Affiliations:** 1 BreMarE - Bremen Marine Ecology, Marine Zoology, University of Bremen, Bremen, Germany; 2 Alfred Wegener Institute for Polar and Marine Research, Bremerhaven, Germany; Institute of Marine Research, Norway

## Abstract

Life-cycles of polar herbivorous copepods are characterised by seasonal/ontogenetic vertical migrations and diapause to survive periods of food shortage during the long winter season. However, the triggers of vertical migration and diapause are still far from being understood. In this study, we test the hypothesis that acidic pH and the accumulation of ammonium (NH_4_^+^) in the hemolymph contribute to the control of diapause in certain Antarctic copepod species. In a recent study, it was already hypothesized that the replacement of heavy ions by ammonium is necessary for diapausing copepods to achieve neutral buoyancy at overwintering depth. The current article extends the hypothesis of ammonium-aided buoyancy by highlighting recent findings of low pH values in the hemolymph of diapausing copepods with elevated ammonium concentrations. Since ammonia (NH_3_) is toxic to most organisms, a low hemolymph pH is required to maintain ammonium in the less toxic ionized form (NH_4_^+^). Recognizing that low pH values are a relevant factor reducing metabolic rate in other marine invertebrates, the low pH values found in overwintering copepods might not only be a precondition for ammonium accumulation, but in addition, it may insure metabolic depression throughout diapause.

## Introduction

Herbivorous copepods are greatly affected by the distinct fluctuation of primary production in polar waters [1]. Certain copepod species have developed species-specific strategies in order to exploit peaks of phytoplankton production in spring and summer and to survive periods of food shortage during the winter season [2-4]. *Calanoides acutus* belongs to the most dominant species with regard to total zooplankton biomass in the Southern Ocean [5], and it is the only Antarctic copepod definitely known to conduct extensive seasonal ontogenetic vertical migrations to survive the food-limited winter season inactively [3]. The late copepodite stages CIV migrate to depths ≥ 500 m at the end of autumn. They further develop into copepodite stage CV at depth and pass into a dormant/resting stage termed diapause, which is characterized by a reduced metabolism in order to conserve energy throughout winter [6,7]. Energy requirements are supplied by massive internal lipid stores (up to 52% of dry mass, [8]), accumulated in the previous productive season [6]. These lipid stores also fuel the restart of development, maturation, and reproduction in spring and influence the physical density of the copepod. The maturation into adult females or males as well as mating itself takes place at the end of winter still at depth. Fertilized females re-ascend to the surface in spring to release their offspring in a time when feeding conditions are optimal [9-11]. Thus, a successful overwintering and reproduction in the following spring can only be achieved, if diapausing copepods remain neutrally buoyant in a relatively stable depth layer where they will not deplete their energy reserves through swimming activities, nor attract potential predators.

In a recent study, Sartoris et al. [12] suggested that the ammonium concentration in the hemolymph of Antarctic copepods plays a critical role for adjusting neutral buoyancy during diapause. *C. acutus* and *Rhincalanus gigas* are the only two Antarctic copepod species where highly elevated ammonium (NH_4_^+^) concentrations of up to 450 mmol L^-1^ have been observed in their hemolymph, whereas none of the other investigated species showed similar results. The authors hypothesized that the replacement of heavier ions (i.e. Na^+^, Mg^2+^, Ca^2+^) by lighter ammonium is necessary to control the overall density of the copepod at overwintering depth [12]. The reduction of physical density by ion replacement and ammonium accumulation is a well-known buoyancy regulation mechanism in a variety of ammoniacal organisms such as the majority of pelagic cephalopods [13,14], deep-sea shrimp *Notostomus gibbosus* [15] and many phytoplankton cells [16], although it has never been reported for copepods before. For most organisms, ammonia (NH_3_) is highly toxic and the concentration in body fluids is typically low [17]. In aqueous solution, ammonium exists as either ammonium ions (NH_4_^+^) or molecular ammonia (NH_3_), with the toxicity strongly dependent upon the pH of the solution. As pH increases, the equilibrium shifts towards the more toxic, un-ionized ammonia (NH_3_) [18-20]. Therefore, we predict a low extracellular pH (pH_e_) in the hemolymph of copepods with elevated ammonium concentrations to avoid the toxicity of ammonia, similar to the ammonium-rich fluids in other ammoniacal marine invertebrates [15,21].

Four Antarctic copepod species as representatives of different life-cycle strategies were studied: *C. acutus*, *Calanus propinquus*, *R. gigas*, and *Paraeuchaeta antarctica*. Although inhabiting the same environment, they show considerable species-specific differences in terms of behavioral, physiological and lipid-biochemical properties. The epipelagic copepods *C. acutus*, *C. propinquus* and *R. gigas* are predominantly small-particle grazers, feeding on phytoplankton and protozoans [22,23]. *C. acutus* is the only diapausing species in the Southern Ocean, whereas information on the behavior of *R. gigas* is less clear. The majority of the *C. propinquus* population remains active in the upper or mid-water layers throughout the whole year, switching to a more omnivorous diet during winter [24,25]. *P. antarctica* is a carnivorous species and, therefore, capable of overwintering without a resting stage, feeding year-round on smaller copepods and other zooplankton [26,27]. 

The current publication extends the hypothesis of ammonium-aided buoyancy in diapausing copepods by postulating that high ammonium concentrations in the hemolymph coincide with low pH values in order to avoid toxic effects of ammonia. For that purpose, a new method providing pH measurements in small volumes (≥ 500 nL) of copepod hemolymph was developed. It is further discussed whether a pH reduction may also be considered as a possible factor that causes the initiation of metabolic depression. In order to test a direct relation between ammonium accumulation and pH regulation to diapause, diapausing and non-diapausing species were studied and compared in two different seasons. 

## Materials and Methods

### Ethics Statement

The present study on planktonic copepods does not include protected or endangered species.

Field work and sampling in the Southern Ocean have been approved by the German Federal Environment Agency (Umweltbundesamt, UBA) as the responsible German national authority according to the national “Act Implementing the Protocol of Environmental Protection to the Antarctic Treaty”.

### Sampling and sorting

**Figure 1:**
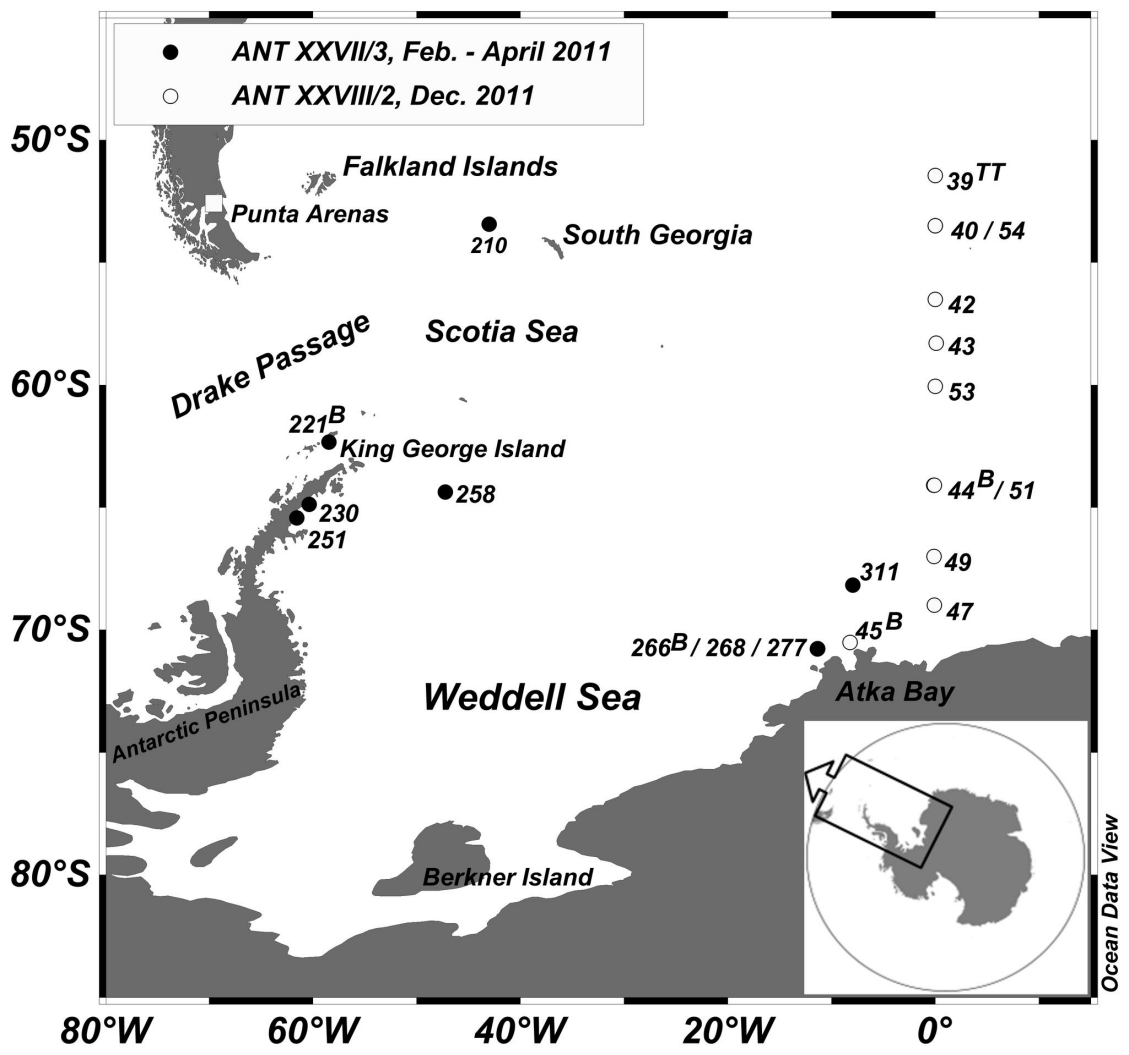
Study area with sampling sites. Study area with sampling sites (ANT XXVII/3: black dots; ANT XXVIII/2: open circles). Letters indicate additional tows with a Bongo net (B) or a Tucker Trawl (TT).

Copepod sampling, pH measurements and experiments were conducted onboard R/V Polarstern on two separate cruises in early austral autumn (ANT XXVII/3, February to April 2011) and late spring (ANT XXVIII/2, December 2011). Samples were collected at a total of 20 stations south of the Antarctic Polar front (APF), except Station 210 west of South Georgia. The exact positions are shown in Figure 1. 

Hydrographical data were recorded prior to any zooplankton haul using a Conductivity-Temperature-Depth (CTD) profiler (SBE 911plus). At each station, stratified depth samples were collected with a vertical haul from a maximum depth of 2000 m to the surface using multiple/opening closing nets (Multinet Maxi, mouth opening 0.5 m^2^; Multinet Midi, mouth opening 0.25 m^2^; 100 µm mesh size for both). Vertical hauls took a maximum of two hours. Up to nine discrete depth strata chosen according to the stratification of the water column were sampled. A flowmeter was attached to the net and used to calculate the volume of water filtered. Additional tows from a maximum depth of 300 m to the surface were conducted by a Bongo net (mouth opening 0.28 m^2^, mesh size 100 μm) and Tucker Trawl (mouth opening 2.25 m^2^, mesh size 1500 μm) to provide supplementary material for biochemical analyses and experiments. Immediately after capture, all copepods were removed from the cod end of the nets and eventually sorted by species, sex and developmental stage. All copepodite stages of *C. acutus*, *R. gigas*, *C. propinquus* and *P. antarctica* were kept separately in jars filled with filtered seawater in temperature-controlled refrigerators or in a cooling container at 0°C for a maximum of two hours previous to hemolymph extraction. The remaining zooplankton from each sample was fixed in 4% borax-buffered formaldehyde in seawater solution for later analyses of community structure.

### Hemolymph extraction and analysis

Individual copepods were transferred to a Petri dish kept on an ice bed and dried carefully with a fuzz-free tissue to remove all remaining seawater. Hemolymph was extracted manually under a dissecting microscope using borosilicate glass capillaries, which were prepared prior to extraction with an electrode puller providing ultra-fine tips. Each hemolymph sample was diluted in 40 µL of de-ionized water and kept in a deep-freezer at -80°C. The cation composition was analyzed by ion chromatography with a DIONEX ICS 2000 at 40°C using an IonPac CS 16 column with methane sulfonic acid (MSA, 30 mmol L^-1^) as an eluent at 0.36 mL min^-1^ flow rate. Inorganic ions such as NH_4_^+^, Na^+^, Mg^2+^, K^+^, and Ca^2+^ were measured and peaks were identified according to retention times in comparison to a cation standard of known composition (Dionex, Six Cation Standard). Cation concentrations are presented as mmol L^-1^. 

### pH_e_-measurements

At least 500 nL of a hemolymph sample were used to measure pH directly onboard using a NanoDrop 3300 fluorometer (Thermo Fischer) and HPTS (8-Hydroxypyrene-1,3,6-trisulfonic acid trisodium salt) as a pH indicator. After sampling a minimum hemolymph volume of 0.5 μL in a pipette (0.1-2 μL), 5% per volume of a HTPS stock solution (50nM HPTS) was added into the pipette. The final HPTS concentration was about 1nM in all measurements. Fluorescence ratios were calculated by dividing the relative fluorescence resulting from 365 nm excitation by the relative fluorescence resulting from excitation at 470 nm (365:470 ratio). pH was calculated using a calibration curve with 50 mM Imidazole (Sigma-Aldrich, Steinheim, Germany) buffered seawater in the pH range from 5.0 to 8.5.

During ANT XXVII/3, pH measurements were conducted at ambient room temperature. Temperature profiles derived from the CTD were used to determine in situ temperatures and results were adjusted according to Ben-Yaakov [28] (temperature coefficient (∆pH/°C ^≈^ 0.01)). During ANT XXVIII/2, pH- measurements were carried out in a temperature-controlled laboratory at in situ temperatures. Measurements resulting in units lower than pH 5.5 and above 8.0 should be interpreted with caution, since depending on the characteristics of the fluorescent dye HPTS error increases beyond these pH values. 

### Statistical analysis

All analyses were performed with R software version 2.14.2. Data were tested for normality with the Shapiro-Wilk-test. According to the distribution, a two-tailed unpaired t-test (confidence interval 95%) or a non-parametric Wilcoxon`s rank-sum test was applied to detect differences between two groups in pH_e_ and cation concentration. For the comparison of more than one means, either a one-way ANOVA or the non-parametric Kruskal-Vallis test was adopted. To analyze the effect of different pH values on the ammonium content in the hemolymph, a linear regression analyses was performed. 

## Results

### Cation composition

**Figure 2:**
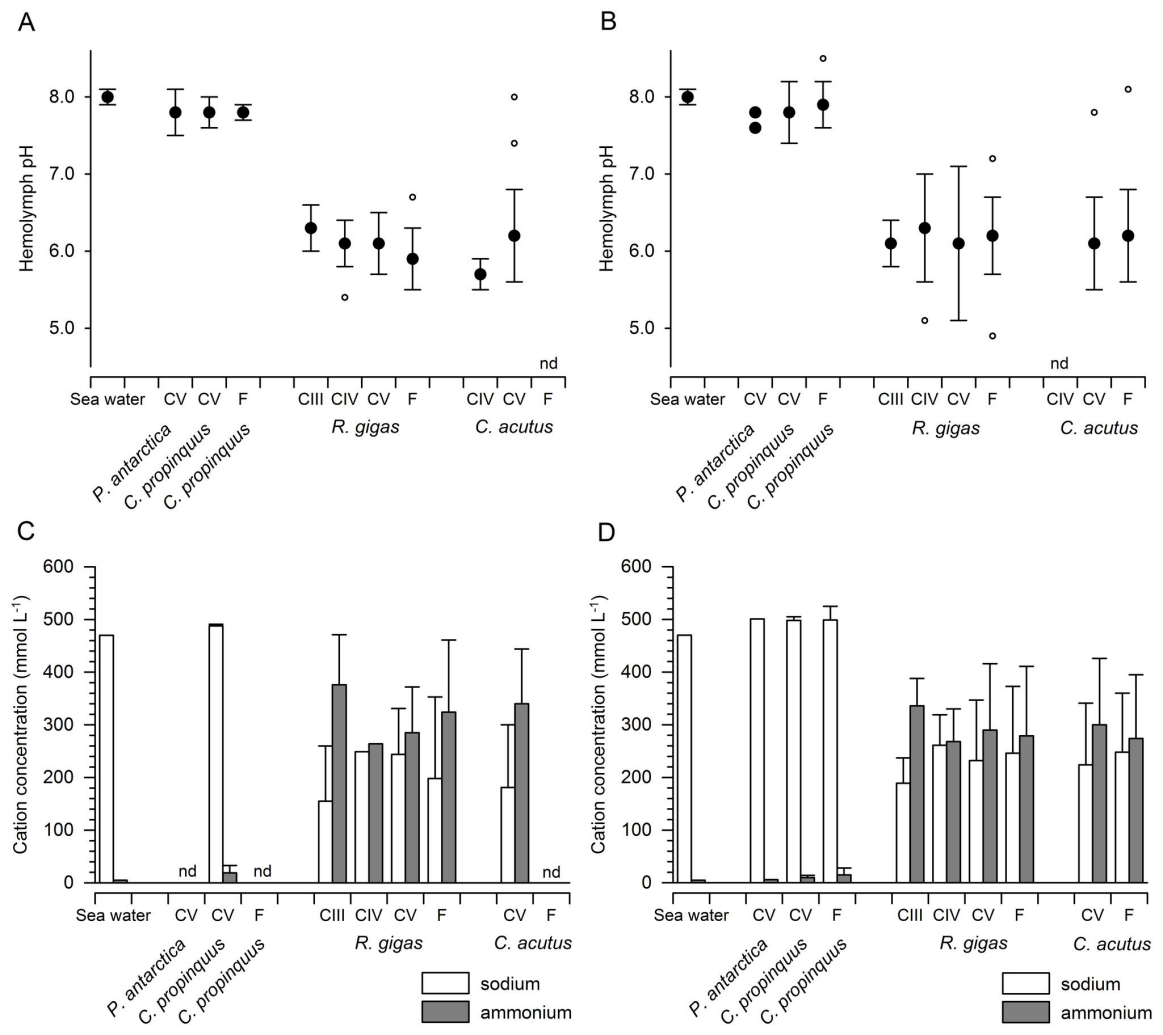
pH values and concentration of sodium and ammonium in seawater and hemolymph of Antarctic copepods. pH values (mean ± standard deviation; A and B, outlying results (more than 1.5 times the interquantile distance away) are represented by open circles) and concentration of sodium and ammonium (mmol L^-1^; C and D; error bars reflect standard deviation) in seawater and in the hemolymph of Antarctic copepods from two different sampling periods (Feb. - April: A and C; December: B and D). Nd not determined, CIII – CV copepodite stages 3 - 5, F females. Number of replicates as in Table 1.

In the hemolymph of the non-diapausing copepods *P. antarctica* and *C. propinquus*, a cation composition almost similar to the ionic composition of seawater was measured, although low levels of up to 48 mmol L^-1^ NH_4_^+^ occurred and Mg^2+^ values were partially reduced to a minimum of 3 mmol L^-1^ (Table 1). Highly elevated concentrations of as much as 515 mmol L^-1^ NH_4_^+^ and greatly reduced levels of down to 15 mmol L^-1^ Na^+^ relative to an average seawater concentration of 470 mmol L^-1^ were present only in the hemolymph of *C. acutus* and *R. gigas* (Table 1, Figure 2). The Na^+^ concentrations in the hemolymph were up to 97% lower than in seawater and explained most of the cation replacement. Additionally, divalent ions such as Mg^2+^ and Ca^2+^ were reduced. In *R. gigas*, the highest individual ammonium concentration was found in stage CIII between February and April (512 mmol L^-1^, Table 1). The lowest individual ammonium concentration of all samples was determined in *C. acutus* CV (9 mmol L^-1^, Table 1) from the surface layer (0 - 50 m) in December. This sample was also characterized by the highest pH value (pH_e_ 7.8, Table 1) measured for *C. acutus* CV during the corresponding sampling period. In contrast, the highest individual ammonium concentration within this species occurred in the hemolymph of CV (515 mmol L^-1^, Table 1) from the deepest layer (2000 - 1500 m). 

**Table 1:** pH and cation composition (mmol L^-1^) in the hemolymph of Antarctic copepod species in two different sampling periods (mean values ± s.d., with their range in parentheses), with values of seawater (cation composition derived from Prosser [51]) for comparison.

Species/		Sampling	pH_e_		Cation composition (mmol L^-1^)					
Source	Stage	Period		n	Na^+^	NH_4_^+^	K^+^	Mg^2+^	Ca^2+^	n
Seawater	na	Austral Spring	8.0 ± 0.1 (7.8 - 8.3)	67	470	0	10	54	10	na
*P. antarctica*	CV	Austral Autumn	7.8 ± 0.3 (7.5 - 8.0)	3						nd
		Austral Spring	7.8	2	524	6	6	4	10	2
			7.6		478	6	10	45	11	
*C. propinquus*	CV	Austral Autumn	7.8 ± 0.2 (7.4 - 8.1)	16	488 ± 14 (468 - 499)	19 ± 3 (16 - 24)	17 ± 4 (12 - 21)	18 ± 8 (11 - 28)	9 ± 0.3 (8.7 - 9.3)	4
		Austral Spring	7.8 ± 0.4 (7.4 - 8.3)	6	498 ± 7 (491 - 504)	10 ± 4 (6 - 14)	19 ± 1 (18 - 20)	14 ± 4 (10 - 18)	9 ± 0.1 (8.8 - 9.1)	3
	F	Austral Autumn	7.8 ± 0.1 (7.6 - 7.9)	4						
		Austral Spring	7.9 ± 0.3 (7.5 - 8.5)	15	499 ± 26 (463 - 523)	15 ± 13 (6 - 48)	9 ± 3 (5 - 16)	16 ± 21 (3 - 54)	10 ± 1 (9 - 12)	9
*R. gigas*	CIII	Austral Autumn	6.3 ± 0.3 (5.7 - 6.7)	10	155 ± 95 (31 - 332)	376 ± 105 (176 - 512)	8 ± 4 (4 - 16)	9 ± 8 (1 - 23)	3 ± 2 (1 - 6)	10
		Austral Spring	6.1 ± 0.3 (5.7 - 6.6)	5	189 ± 48 (124 - 227)	336 ± 52 (291 - 407)	7 ± 2 (6 - 9)	11 ± 3 (7 - 15)	6 ± 2 (3 - 9)	4
	CIV	Austral Autumn	6.1 ± 0.3 (5.4 - 6.4)	8	249	264	19	13	6	1
		Austral Spring	6.3 ± 0.7 (5.1 - 7.3)	7	261 ± 58 (166 - 340)	268 ± 62 (188 - 375)	6 ± 3 (2 - 9)	9 ± 3 (2 - 12)	5 ± 1 (5 - 6)	7
	CV	Austral Autumn	6.1 ± 0.4 (5.4 - 6.8)	22	244 ± 87 (166 - 344)	285 ± 87 (184 - 356)	10 ± 7 (3 - 18)	8 ± 4 (2 - 11)	3 ± 2 (1 - 5)	5
		Austral Spring	6.1 ± 1.0 (4.8 - 7.0)	4	232 ± 115 (90 - 360)	290 ± 126 (159 - 452)	11 ± 6 (5 - 20)	13 ± 8 (3 - 26)	4 ± 2 (1 - 7)	5
	F	Austral Autumn	5.9 ± 0.4 (5.1 - 6.7)	17	198 ± 137 (55 - 427)	324 ± 155 (50 - 484)	11 ± 3 (5 - 15)	14 ± 16 (2 - 49)	3 ± 3 (0.2 - 9)	9
		Austral Spring	6.2 ± 0.5 (4.9 - 7.2)	24	246 ± 127 (15 - 465)	278 ± 131 (52 - 502)	9 ± 4 (3 - 18)	10 ± 7 (2 - 23)	5 ± 3 (1 - 11)	25
*C. acutus*	CIV	Austral Autumn	5.7 ± 0.2 (5.4 - 6.0)	5						nd
	CV	Austral Autumn	6.2 ± 0.6 (5.4 - 8.0)	23	181 ± 104 (28 - 427)	340 ± 119 (48 - 512)	12 ± 4 (6 - 21)	13 ± 12 (1 - 49)	4 ± 3 (0.03 - 11)	32
		Austral Spring	6.1 ± 0.6 (4.8 - 7.8)	59	224 ± 117 (30 - 514)	300 ± 126 (9 - 515)	9 ± 5 (1 - 20)	12 ± 9 (1 - 35)	5 ± 3 (1 - 10)	45
	F	Austral Spring	6.2 ± 0.6 (5.1 - 8.1)	56	248 ± 112 (56 - 400)	274 ± 121 (110 - 469)	11 ± 5 (1 - 20)	11 ± 9 (2 - 31)	6 ± 3 (2 - 11)	25

Dashed line separates non-resting (above) from resting species (below). copepodite stages 3 - 5 (CIII - CV), F females¸ n number of analyzed individuals, na not applicable, nd not determined

### pH of hemolymph

In the hemolymph of the non-diapausing species *P. antarctica* and *C. propinquus*, mean pH values of 7.8 ± 0.3 were measured in both seasons (Table 1, Figure 2). Individual measurements were never lower than pH 7.4 in both species, seasons and all developmental stages (Table 1, Figure 2). 

Lower pH levels only occurred in the hemolymph of diapausing *C. acutus* and *R. gigas*, and differences in the mean hemolymph pH were statistically discernible between diapausing (*C. acutus* and *R. gigas*) and non-diapausing (*P. antarctica* and *C. propinquus*) species (ANOVA, p<0.0001). In *R. gigas*, mean hemolymph pH ranged from 5.9 to 6.3 in both seasons (Table 1, Figure 2). Individual measurements did not exceed pH 6.8 from February to April, while individual values higher than pH 7 were detected in December (*R. gigas* F: 7.2; CIV: 7.3, Table 1). 

In *C. acutus*, mean hemolymph pH varied from 5.7 to 6.2 between February and April and 6.1 to 6.2 in December (Table 1, Figure 2). The overall highest hemolymph pH was measured in a *C. acutus* female (pH_e_ 8.1, Table 1) from the surface layer (100 - 0 m) in December. Overall means (without division into developmental stages) between February and April were pH 6.1 ± 0.4 in *R. gigas* (n = 57) and pH 6.1 ± 0.6 in *C. acutus* (n = 28). In December the overall mean amounted to pH 6.2 ± 0.6 for both species (*R. gigas* n = 40, *C. acutus* n = 115). 

**Figure 3:**
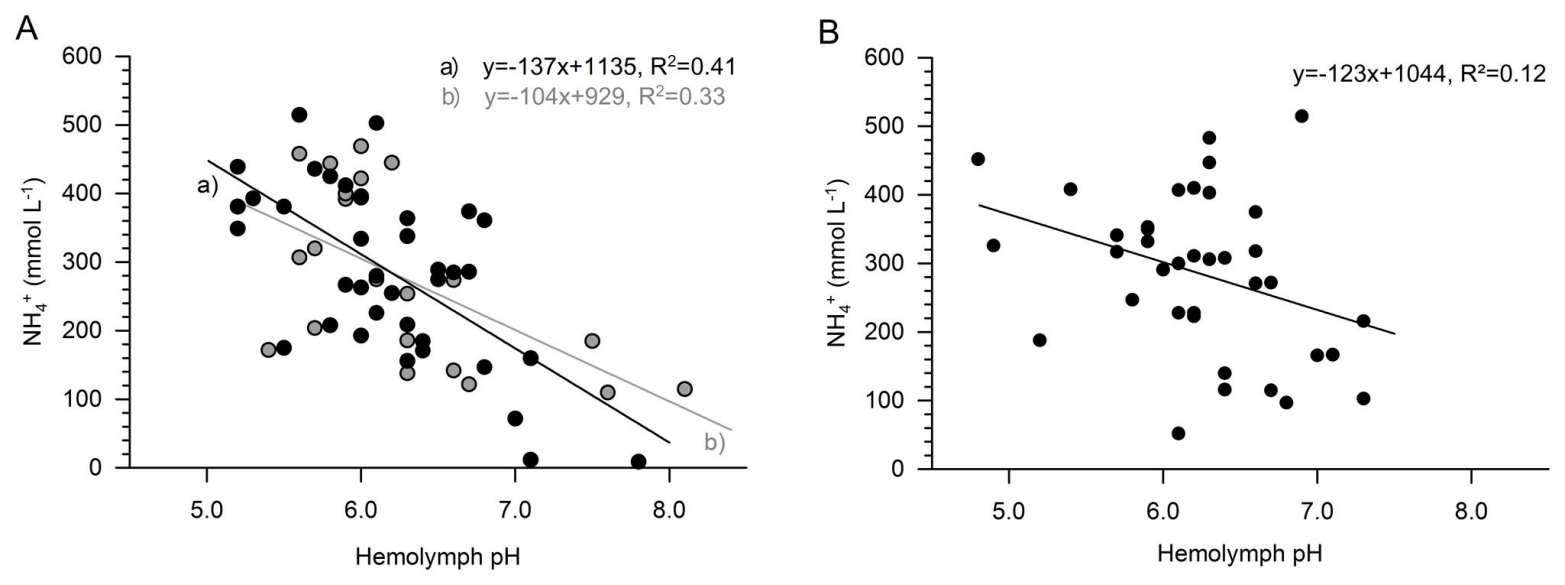
Relationship between pH and concentration of ammonium (NH_4_^+^). Relationship between pH and concentration of ammonium (NH_4_^+^, mmol L^-1^) in the hemolymph of *C. acutus* (A; copepodite stage CV (a) y=-137x+1135, R^2^=0.41, p<0.01; females (b) y=-104x+929, R^2^=0.33, p<0.01) and *R. gigas* (B; all stages combined, y=-123x+1044, R^2^=0.12, p=0.034).

The interaction between pH and ammonium concentration in the hemolymph of *C. acutus* and *R. gigas* was tested for the expedition in December (Figure 3). The ammonium content in the hemolymph was significantly affected by acidity and increased with lower pH values in both species. The correlation was most pronounced in *C. acutus* CV (y=-137x+1135, R^2^=0.41, p<0.01), followed by *C. acutus* females (y=-104x+929, R^2^=0.33, p<0.01). In *R. gigas*, the correlation was less pronounced, but still discernible (all stages combined: y=-123x+1044, R^2^=0.12, p=0.034) (Figure 3).

## Discussion

The overall physical density of a diapausing copepod at depth must equal the density of the surrounding seawater to provide a stable position in the water column and to avoid a depletion of energy reserves for swimming activities. Proteins and the exoskeleton (1080 - 1240 kg m^-3^ in boreal-Atlantic diapausing *Calanus finmarchicus* [29]), exceed the density of seawater (^≈^ 1037.4 kg m^-3^ at 0°C temperature, 35 psu salinity and overwintering depth of 2000 m, calculation based on Fofonoff and Millard [30]). Thus, diapausing copepods must compensate the down-force by accumulating less dense body components that provide uplift and help maintain an overall neutral buoyancy. 

An increasing number of studies have focused on the central role of low-density lipids in both regulating buoyancy and determining overwintering depths in diapausing copepods [10,29,31-35]. Only recently, special emphasis has been placed on the exact composition and degree of unsaturation of the stored wax esters. Wax esters are assumed to undergo phase transitions from liquid to solid state at high water pressures typical of overwintering depths and low temperatures. Such phase transitions could favorably increase the overall density of copepods and facilitate neutral buoyancy at depth [33-35]. However, notwithstanding the importance of lipid deposits as energy reserves, they are rather counterproductive in the course of downward migration, since copepods start to descend at the end of the productive season when lipid contents are at their maximum and hence, lipid-regulated buoyancy is high. In addition, Campbell and Dower [10] showed that lipid-based buoyancy is rather unstable and difficult to regulate, since small changes in lipid content will have dramatic consequences for buoyancy. 

### Ammonium-aided buoyancy

The presence of elevated ammonium concentrations within the hemolymph of only copepods known to undergo diapause in winter was confirmed in this study (Table 1; see 12 for review of previously measured values). Simultaneously, Na^+^ and to a lesser extent Mg^2+^ and Ca^2+^ concentrations were reduced in relation to both seawater and the hemolymph of non-diapausing copepods. Variable amounts of K^+^ are not further discussed in this study, since elevated concentrations are most likely the result of injured body tissue in the course of the hemolymph extraction, allowing the leakage of K^+^ from the intracellular into the extracellular space. 

Our findings of cation replacement in the hemolymph of diapausing copepods are in good accordance with changes in the ion composition in body fluids from a range of marine organisms known to use low-density fluids for buoyancy regulation [13,15,16,21]. For comparison, concentration of NH_4_^+^ in the deep-sea shrimp *N. gibbosus* was 296 ± 51 mmol L^-1^ in the carapace fluid and 217 ± 54 mmol L^-1^ in the hemolymph [15].

Compared to the extra cost of swimming or the accumulation of low-density organic compounds such as lipids, the energetic costs involved in the production of ammonium are low, since ammonium is a waste product from the catabolism of proteins and amino acids. Furthermore, in contrast to other buoyancy mechanisms, ammonium-aided buoyancy is independent from high ambient pressures and rapid changes in depth [13] and is therefore well suited for the extensive vertical migrations of copepods.

### pH of hemolymph

Ammonia is highly toxic for most organisms and the concentration in the hemolymph of aquatic crustaceans is generally below 0.8 mmol L^-1^ [17]. In decapod shrimps for instance, 96-h LC_50_ values for adult *Penaeus paulensis* and juvenile *Litopenaeus vannamei* were, respectively, 2.4 mmol L^-1^ [36] and 2.2 mmol L^-1^ total ammonia [37]. The protonation of ammonia to form the comparatively less toxic ionized form NH_4_^+^ is strongly dependent on the H^+^ concentration, and to a lesser extent upon temperature and salinity of the respective solution. At a pH of 7.8, 0°C and 32-40 psu salinity (pK ^≈^ 10.16), 99.6% of total ammonium exists in the ionic form NH_4_^+^, whereas 0.4% is present as NH_3_ [38]. At ammonium concentrations of ^≈^ 500 mmol L^-1^ as measured in the hemolymph of diapausing copepods in this study, 0.4% would result in a NH_3_ concentration of 2 mmol L^-1^, which far exceeds the general hemolymph concentration of ≤ 0.8 mmol L^-1^ in aquatic crustaceans [17]. Under acidic conditions of pH ^≈^ 6.0, as measured in the hemolymph of diapausing copepods with elevated ammonium concentrations, only 0.007% of total ammonia is present as toxic NH_3_ [38], resulting in a concentration of 0.035 mmol L^-1^. Since NH_3_ is lipid-soluble, uncharged and therefore easily diffusible across phospholipid membranes, it is regarded as the most toxic form in fish [18] and aquatic crustaceans [19]. 

To avoid ammonia toxicity, ammoniacal deep-sea crustaceans and squids sequester ammonium fluids in specialized vacuoles, chambers or gelatinous layers [14,15,39]. So far, there is no evidence for the existence of such a cellular unit in copepods. 

The present study revealed very low pH values only in the hemolymph of diapausing copepods (Table 1, Figure 2). Moreover, the amount of ammonium was correlated to the respective hemolymph pH in both overwintering species (Figure 3). *C. acutus* and *R. gigas* had pH values of 6.1 to 6.2 in both seasons. These results are comparable to the pH in the ammonium-rich carapace fluid of *N. gibbosus* (pH 6.6 ± 0.08) [15] and in the vacuolar fluid of ammoniacal squid (≥ 5.1) [21]. At such low pH levels, virtually all ammonium is present in the non-toxic ionized form, which additionally reduces the loss of ammonia by diffusion [39,40].

Marine planktonic crustaceans are generally sensitive to low pH conditions and exposure to elevated H^+^ concentrations can cause high mortality rates in zooplankton communities [41]. In addition, acidic pH conditions in the intracellular milieu have been shown to be relevant factors depressing metabolic rate in a range of other invertebrates during dormancy or environmental hypercapnia [42–44]. Since metabolic depression in diapausing copepods is essential to save energy during the food-limited winter period, the low pH values found in overwintering copepods might be beneficial for a successful implementation of diapause. Indeed, potential benefits of a low extracellular pH leading to a reduced aerobic energy turnover and thus metabolic depression have already been established for the marine worm *Sipunculus nudus* during anaerobiosis [45–47]. 

### Inter-individual/intra-specific variability

Differences in both hemolymph pH and cation composition were discernible between diapausing and non-diapausing species, whereas no statistical differences could be determined between seasons, sampling depths or ontogenetic stages. According to the assumption that ammonium-aided buoyancy changes with season and that hemolymph pH controls metabolic depression, ammonium accumulation should be a seasonal phenomenon with maximum levels in overwintering stages at depth and minimum levels in active stages at the surface. Deviations from this prediction in the present data may be explained by the fact that our samples were collected in the transitional periods of autumn and spring where considerable variation in the individual life histories could not be excluded. Future studies should focus on ammonium concentration and hemolymph pH of Antarctic copepods during the active phase in summer and during diapause in winter.

In *C. acutus*, copepodite stage CV represents the main overwintering cohort. The fact that maximum ammonium concentration in *C. acutus* was observed in copepodite stage CV from the deepest water layer supports the idea that ammonium-aided buoyancy is most important in diapausing stages at overwintering depth. Nevertheless, adults have to ascend to the surface in spring with reduced lipid deposits [4,6]. Thus, elevated ammonium levels and high buoyancy is beneficial for the return to the surface. Ammonium levels and/or excretion rates may vary throughout the water column from individual to individual in relation to other factors such as maturity level or lipid content.

In *R. gigas*, intraspecific variation of hemolymph pH was high and most pronounced in stages CIV and CV in spring. Published data show that most likely not all individuals of *R. gigas* overwinter inactively. Resting stages were found at depth in the marginal ice-zone of the southern Scotia Sea [48], whereas actively feeding and apparently reproducing individuals occurred in the area of the Antarctic Peninsula during winter [49]. Unlike *C. acutus*, *R. gigas* has a more flexible, one- or two-year life cycle, meaning that parts of the population can maintain in the surface waters during winter, while the rest descends and enters diapause [3]. In consequence, we possibly caught and measured a “mixture” of individuals with different physiological backgrounds. Nevertheless, our hypothesis was supported by the observation that the highest ammonium content was measured in copepodite stage CIII in autumn (512 mmol L^-1^, Table 1), which represents one of the main overwintering stages in *R. gigas* [50], whereas minimum concentrations were found in fully developed females (50 - 52 mmol L^-1^, Table 1), which definitely had passed through diapause, molting and maturation. 

In contrast, the correlation between pH and ammonium was stronger in *C. acutus* CV (Figure 3) and is indicative for a clearly defined one-year life cycle in which successful spawning is closely restricted to the beginning of the productive season [3]. 

Accumulation of ammonium and the replacement of ions with a higher solute mass (e.g. Na^+^, Mg^2+^ and Ca^2+^) in the hemolymph of Antarctic copepod species known to undergo diapause in winter was confirmed in this study. These findings support the idea of a relation between vertical ontogenetic migration and ammonia aided buoyancy in Antarctic copepods. Moreover, low pH values were measured only in the hemolymph of diapausing copepods with elevated ammonium levels. The ammonium content was statistically correlated to the respective pH of the hemolymph sample and increased with lower pH values. Further research should focus on the effect of low pH for metabolic depression and its possible role as the sought-after trigger for controlling diapause in Antarctic copepods.
